# Supplementary material for: Cryo-EM structure of a lysozyme-derived amyloid fibril from hereditary amyloidosis
Source: Nat Commun. 2024 Nov 7;15:9648. doi: 10.1038/s41467-024-54091-7 (PMC11543692; doi:10.1038/s41467-024-54091-7)
Supplement: Supplementary file 1 — Supplementary Information [file 41467_2024_54091_MOESM1_ESM.pdf]

Supplementary Information

Cryo-EM structure of a lysozyme-derived amyloid fibril from hereditary amyloidosis

Sara Karimi-Farsijani[1]*, Kartikay Sharma[1], Marijana Ugrina[2], Lukas Kuhn[1], Peter Benedikt Pfeiffer[1], Christian Haupt[1], Sebastian Wiese[3], Ute Hegenbart[4], Stefan O. Schönland[4], Nadine Schwierz[2], Matthias Schmidt[1], Marcus Fändrich[1]

[1] *Institute of Protein Biochemistry, Ulm University, 89081 Ulm, Germany.

[2] Institute of Physics, University of Augsburg, 86159 Augsburg, Germany.

[3]Core Unit Mass Spectrometry and Proteomics, Medical Faculty, Ulm University, 89081, Ulm, Germany.

[4] Medical Department V, Amyloidosis Center, Heidelberg University Hospital, 69120 Heidelberg, Germany.

Corresponding author, S.K. (email: sara.karimi-farsijani@uni-ulm.de)

Supplementary Figures

Supplementary Figure 1

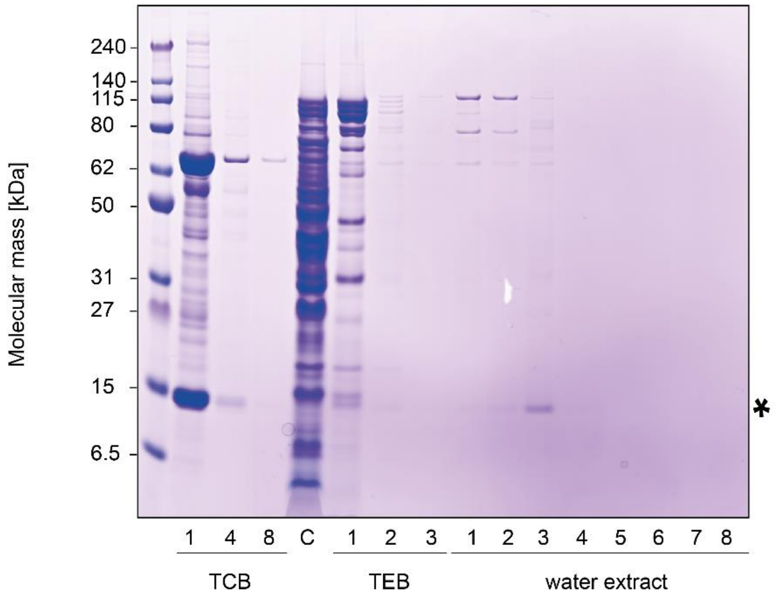

Supplementary Figure 1.

Denaturing polyacrylamide gel of the LysD87G amyloid protein.

Coomassie-stained denaturing protein electrophoresis gel of the extracted ALys amyloid fibrils. TCB: supernatants of the washing steps with Tris calcium buffer; C: pellet after collagenase treatment, TEB: supernatants of the washing steps with Tris EDTA buffer; water extract: supernatants of the water extraction steps, containing the isolated amyloid fibrils. Asterisk: ALys fibril protein. The experiment was performed independently three times with similar results. Source data are provided as a Source Data file.

Supplementary Figure 2

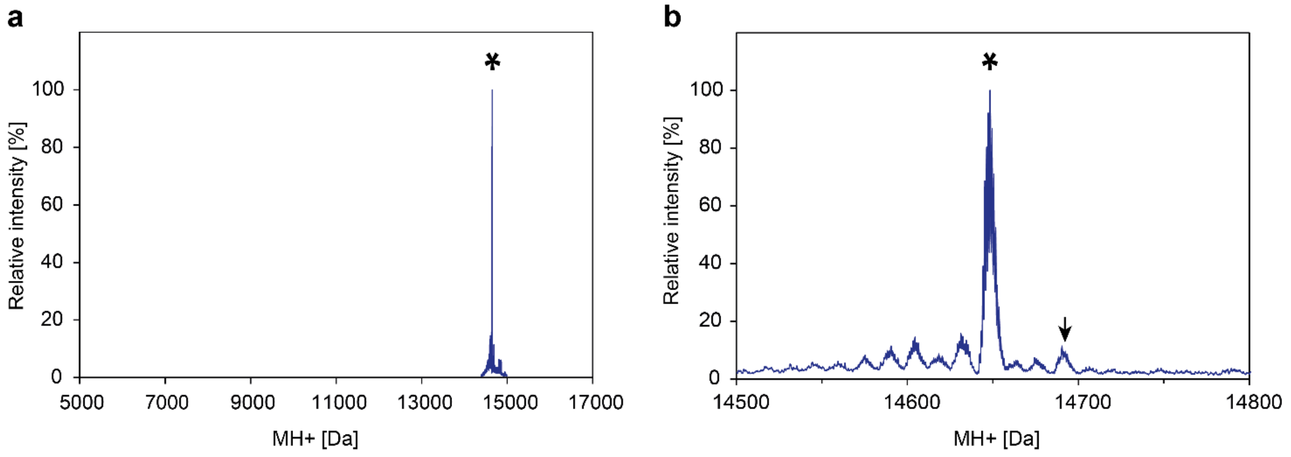

Supplementary Figure 2.

Mass analysis.

(a) Deconvoluted mass spectrum of the ALysD87G amyloid fibril protein (n = 1, biological replicate).

(b) Close up of the spectrum shown in (a). The black star indicates the experimentally determined average mass of the fibril protein (14,648.38 Da). The theoretical mass of D87G human lysozyme is 14,634.47 Da, with four intact disulfide bonds. The black arrow points to the expected position of the WT lysozyme peak based on the calculation of the theoretical mass of 14,692.51 Da, assuming four disulfide bonds.

Supplementary Figure 3

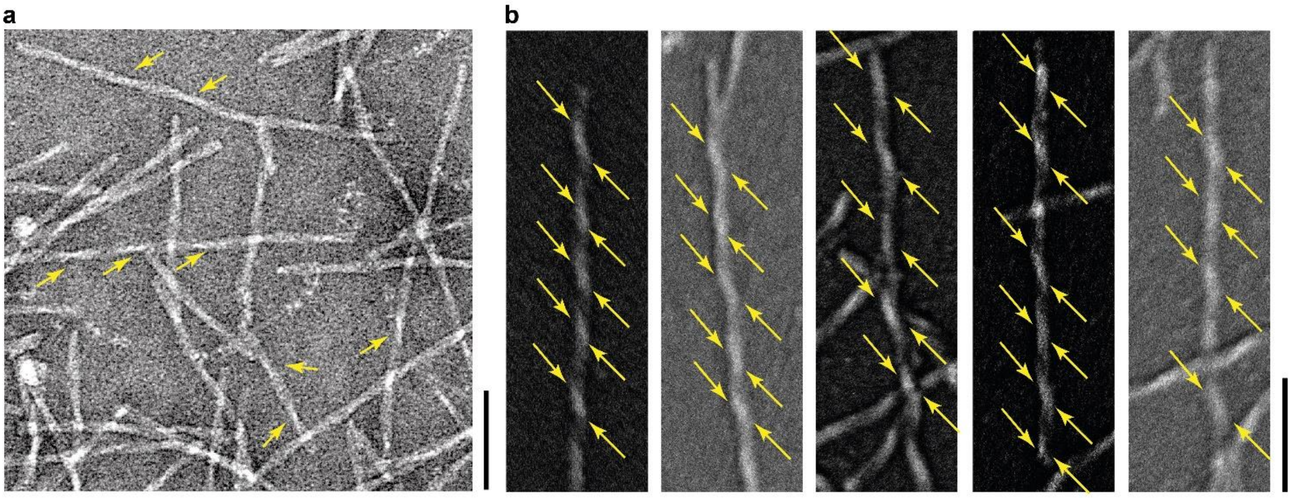

Supplementary Figure 3.

Electron microscopic analysis of ALys amyloid fibrils.

(a) Negatively stained sample of the isolated ALys amyloid fibrils analyzed with TEM. Scale bar: 100 nm. Yellow arrows indicate the cross-overs on some fibrils. (b) SEM images of ALys amyloid fibrils after platinum side shadowing showing the left-hand fibril twist. Yellow arrows are drawn to guide the eye. Scale bar: 100 nm.

Supplementary Figure 4

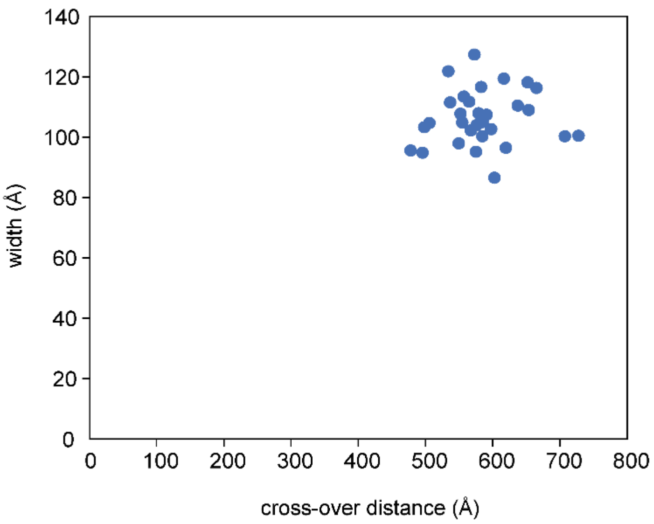

Supplementary Figure 4.

Distribution of the fibril width and cross-over distance.

Analysis of 30 fibrils from cryo-EM images yields mean values of 584 ± 58 Å for the cross-over distance and of 106 ± 9 Å for the fibril width.

Supplementary Figure 5

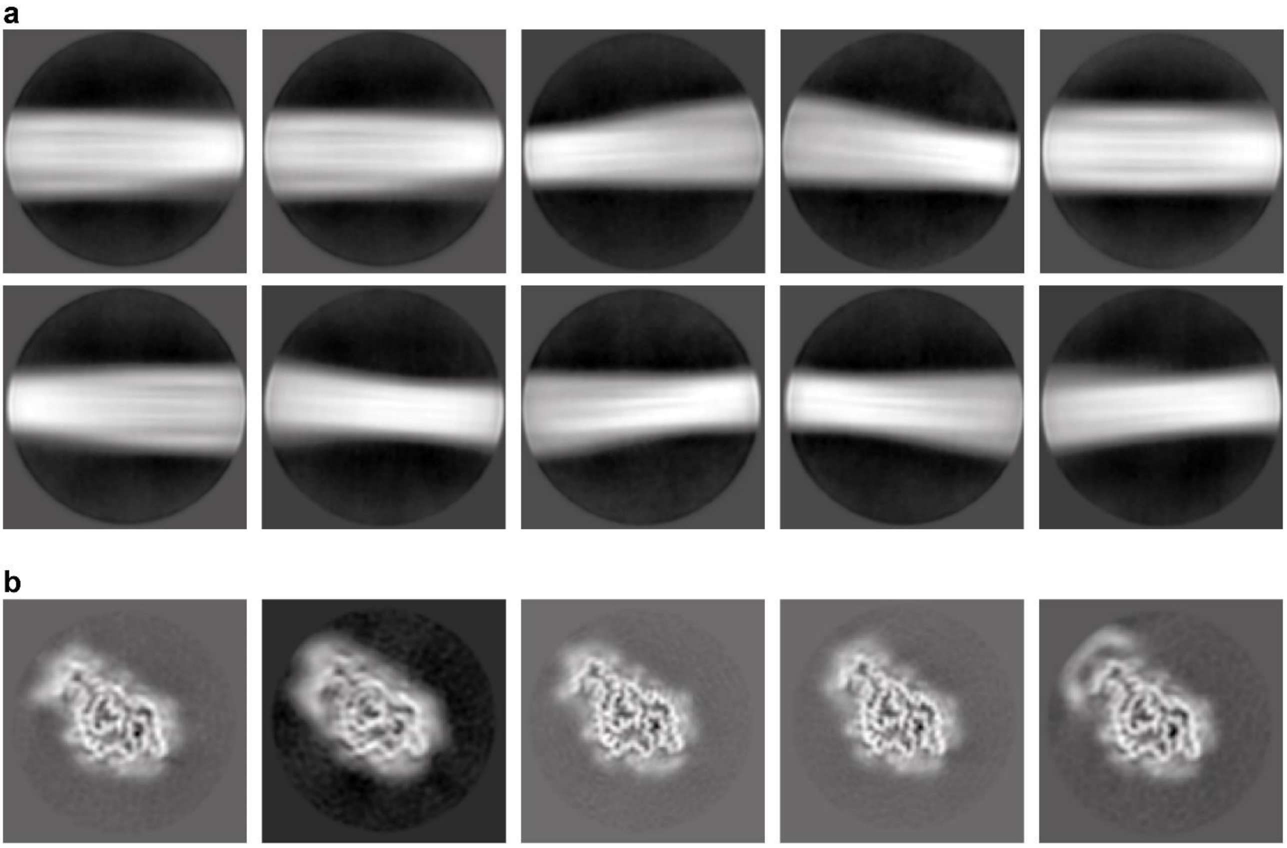

Supplementary Figure 5.

2D and 3D classification results.

(a) 2D class averages obtained with all picked particles after a 2D classification with ten classes. (b) 3D classes obtained with all particles after a 3D classification with five classes. Hence, 2D and 3D classes did not provide evidence for alternative fibril morphologies.

Supplementary Figure 6

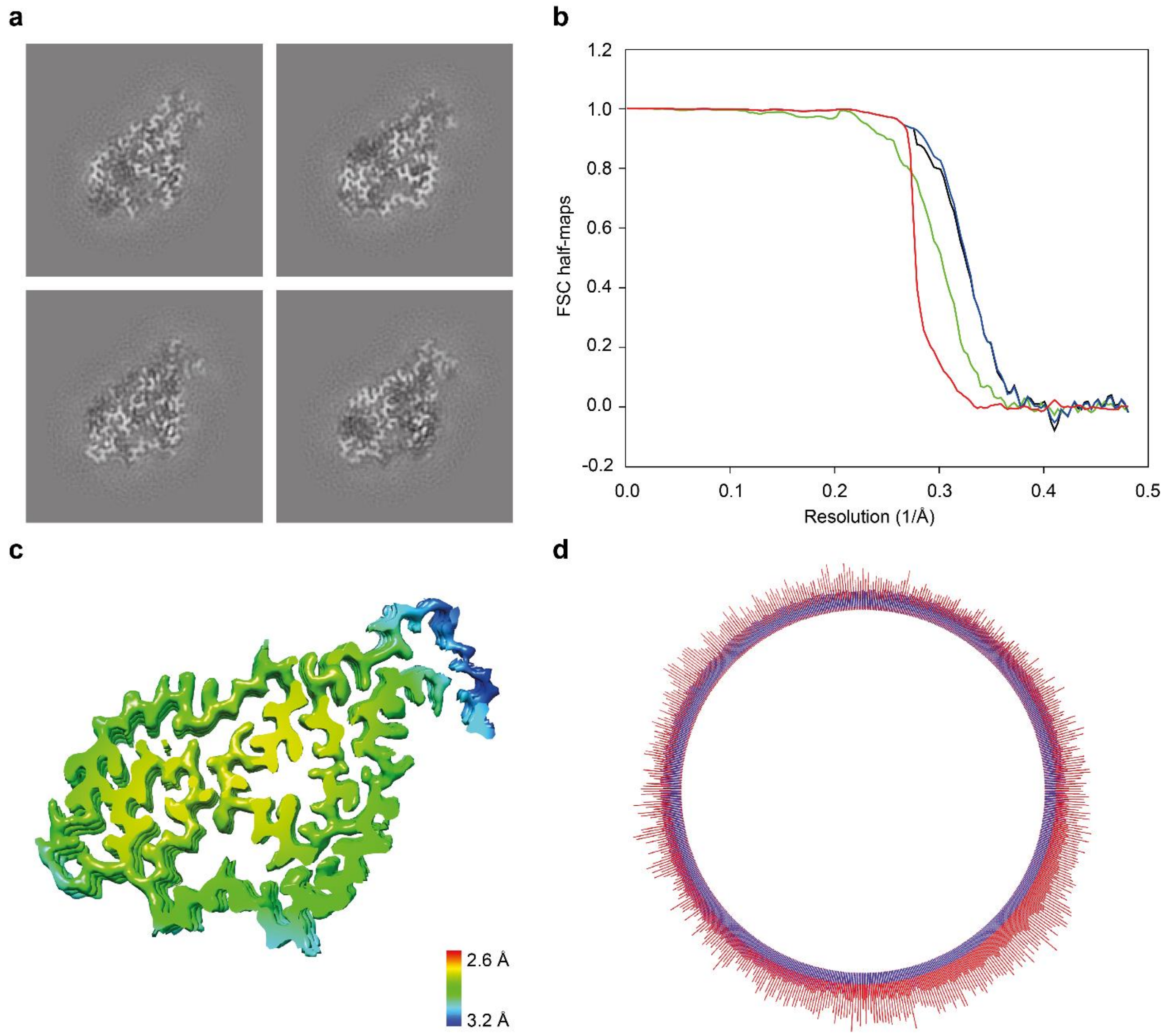

Supplementary Figure 6.

Reconstructed 3D map and corresponding FSC curve.

(a) Images of four consecutive cross-sectional sections of the reconstructed 3D map. Each section is 1.04 Å thick. (b) FSC of the two half maps. Black: FSC corrected; green: FSC unmasked maps; blue: FSC masked maps; red: corrected FSC phase randomized masked maps. (c) Estimation of the local resolution of the 3D map with color key. (d) Illustration of the angular distribution of the particles used for the final reconstruction. Source data are provided as a Source Data file.

Supplementary Figure 7

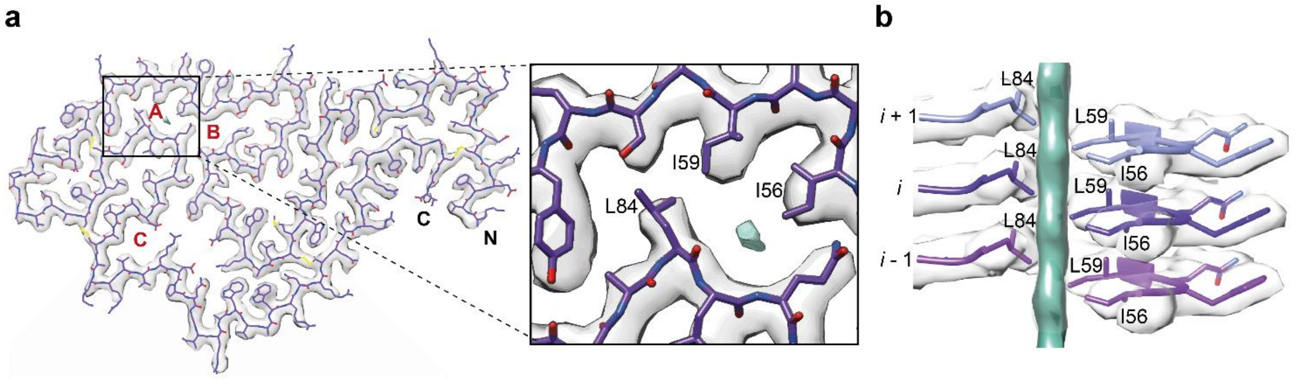

Supplementary Figure 7.

Cavities and inclusions in the fibril structure.

(a) Cross-section of the 3D map (grey) and overlaid with atomic model (purple). The cavities are marked with the red letters A, B and C. The close-up shows the extra density (green) of cavity A. (b) Side view of the extra density (green) of cavity A, which is not fully separated in the direction of the fibril z-axis.

Supplementary Figure 8

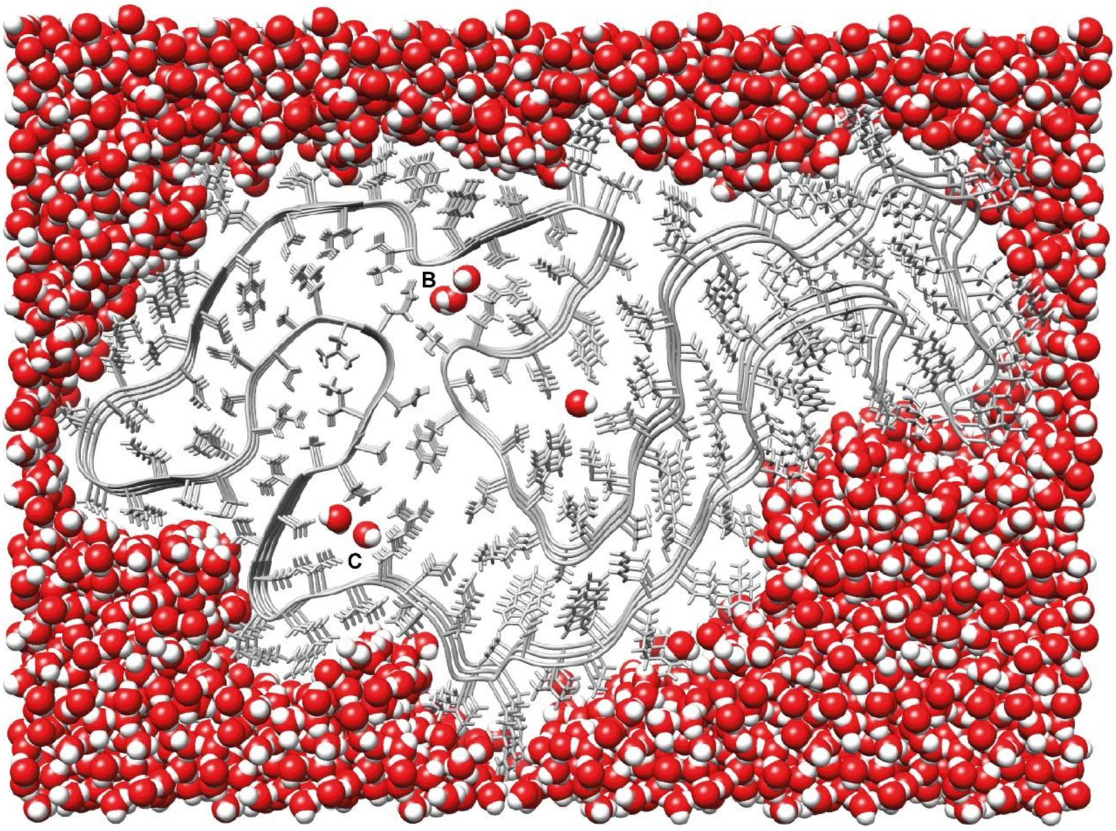

Supplementary Figure 8.

Modelling of the bound water molecules.

View of the central three layers of a nine-layer stack of the fibril (grey) and the corresponding water molecules (red/white), which was generated using Amber tool within Chimera[1]. Note that cavities B and C are able to fit buried water molecules.

Supplementary Figure 9

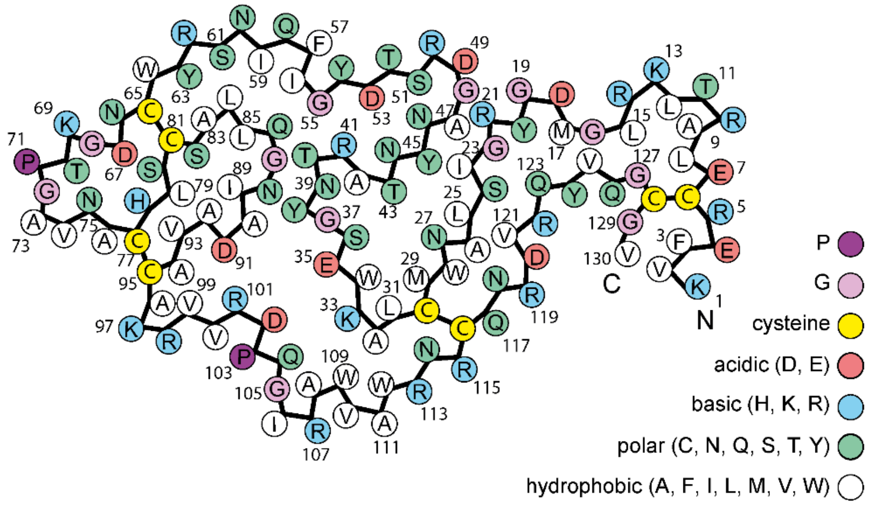

Supplementary Figure 9.

Schematic representation of the fibril protein.

The black zig-zag line refers to the protein backbone and to the disulfide bonds, the circles indicate the amino acid residues. The residues are colored according to their chemical properties.

Supplementary Figure 10

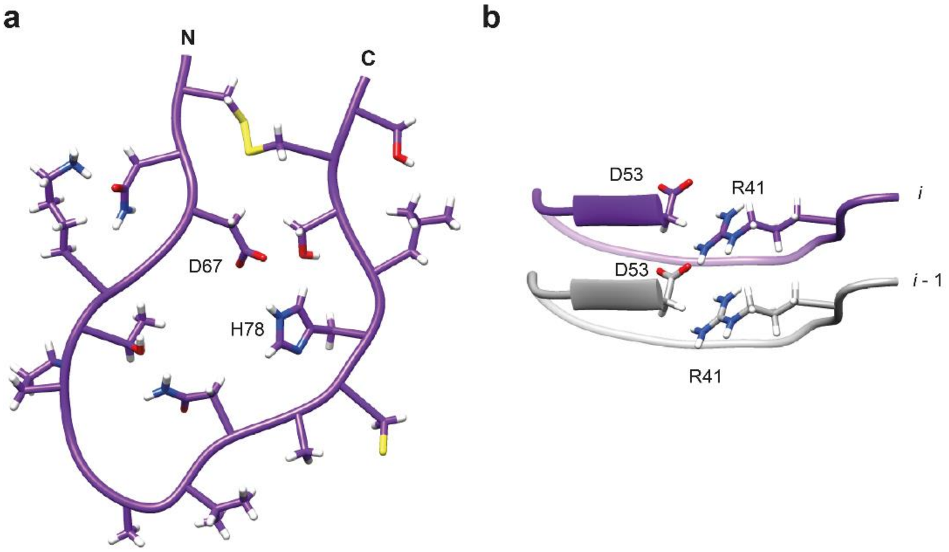

Supplementary Figure 10.

Charge pairs in the ALys amyloid fibril.

(a) Detailed view of the cross-section shows the spatial proximity of the oppositely charged residues Asp67 and His78. (b) Side view of the fibril showing cross-layer interactions between Arg41 (layer *i,* purple) and Asp53 (layer *i* – 1, grey).

Supplementary Figure 11

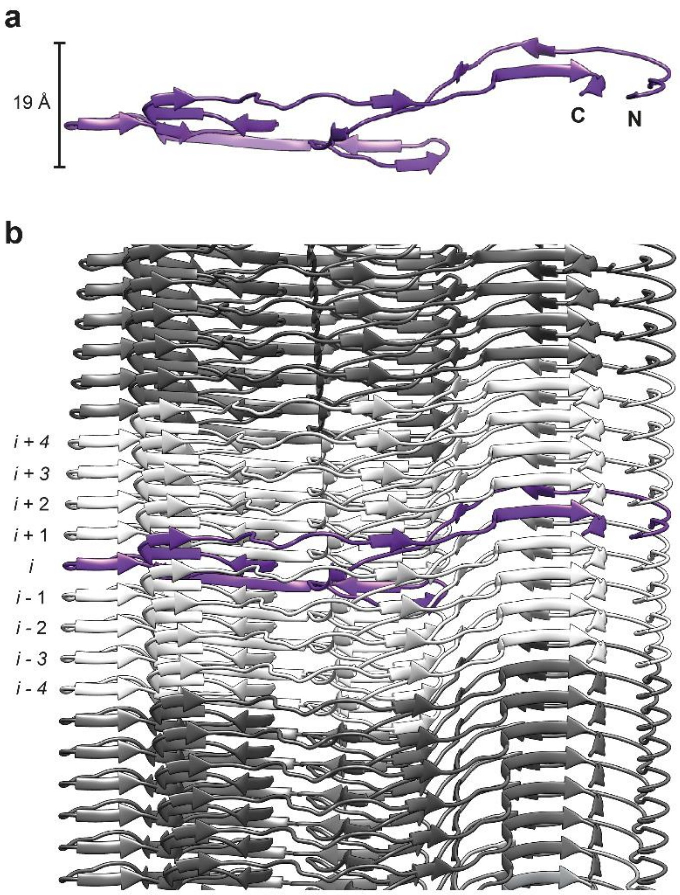

Supplementary Figure 11.

Molecular interactions and z-axial elevation of the fibril protein.

(a) Ribbon diagram of one molecular layer, showing an axial height of the fibril protein of 19 Å, as measured by the Cα atoms of residues Gly19 and Asn44. (b) Side view of the fibril. Eight molecular layers (light grey) form intermolecular interfaces with the central layer (*i*, purple) as calculated by program PDBePISA[2].

Supplementary Figure 12

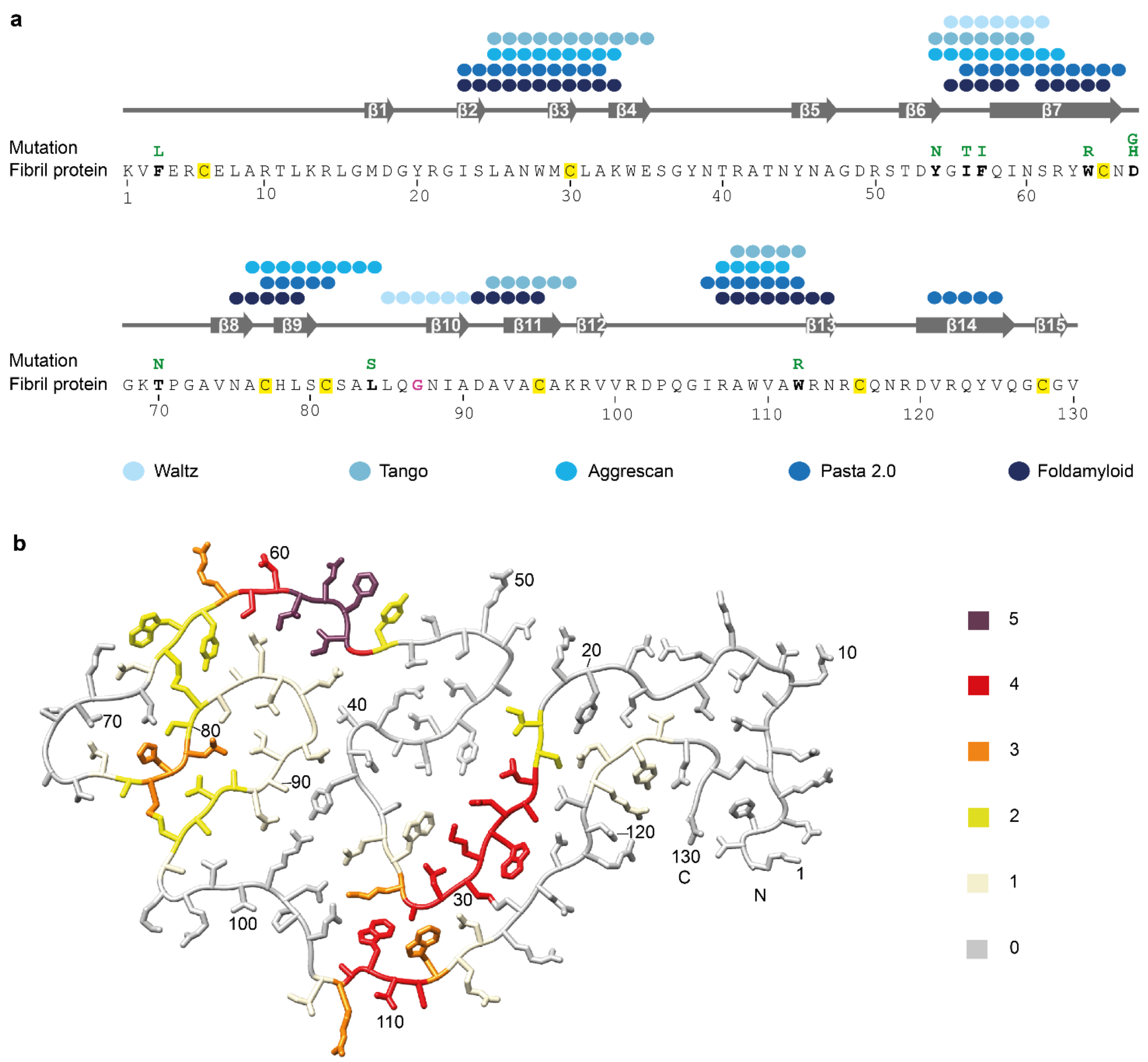

Supplementary Figure 12.

Location of aggregation prone segments in the fibril structure.

(a) The protein sequence of LysD87G and a schematic view of the fibril protein secondary structure (grey). Dots above the sequence indicate residues with a high aggregation tendency according to five different methods, as indicated in the figure. (b) Ribbon diagram of the ALys amyloid fibril structure color coded according to the aggregation score (0-5), as indicated in the figure.

Supplementary Figure 13

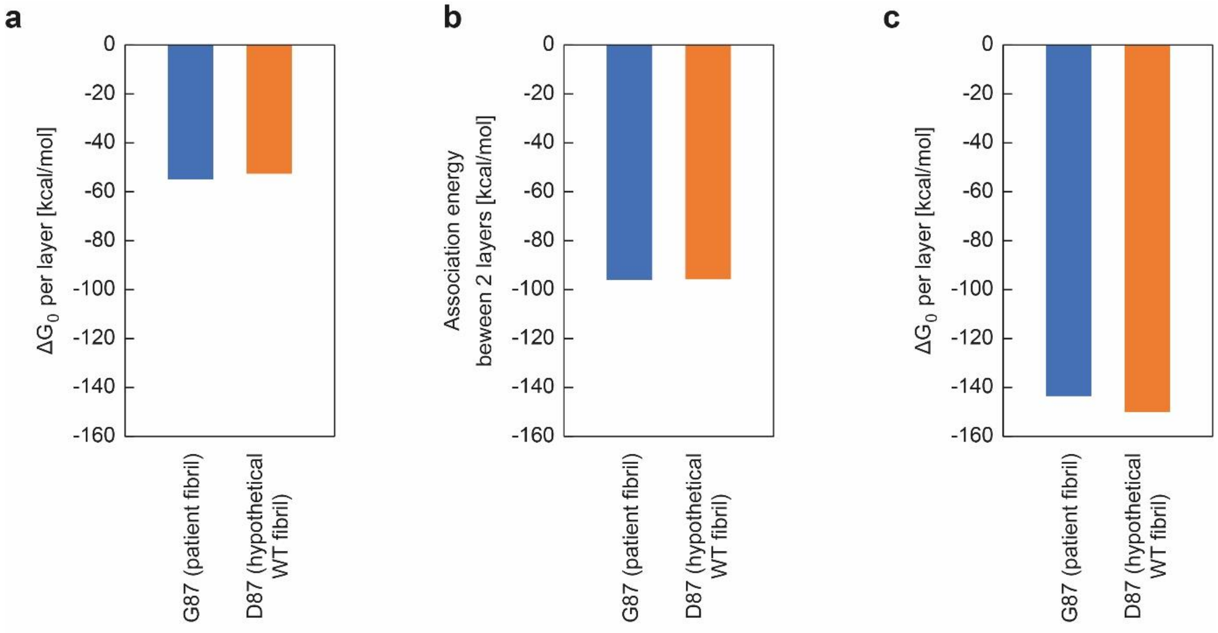

Supplementary Figure 13.

Theoretic stabilities of the patient fibril and a hypothetical WT ALys fibril.

(a-c) Theoretic stability values of the patient fibril, containing a G87 residue, and a hypothetical WT fibril (D87) obtained with the programs Amyloid Illustrator[3] (a), PDBePISA[2] (b) and FoldX[4](c). The Amyloid Illustrator server calculates the free energy per fibril layer. PDBePISA[2] calculates the energy needed to dissociate two adjacent layers of the amyloid fibril, which was inverted to obtain the energy gain upon association. FoldX[4] determines, for one fibril layer, the free energy difference between the folded and unfolded state. To obtain the energy gain upon the formation of the fibril protein fold, the energy values were inverted.

Supplementary Figure 14

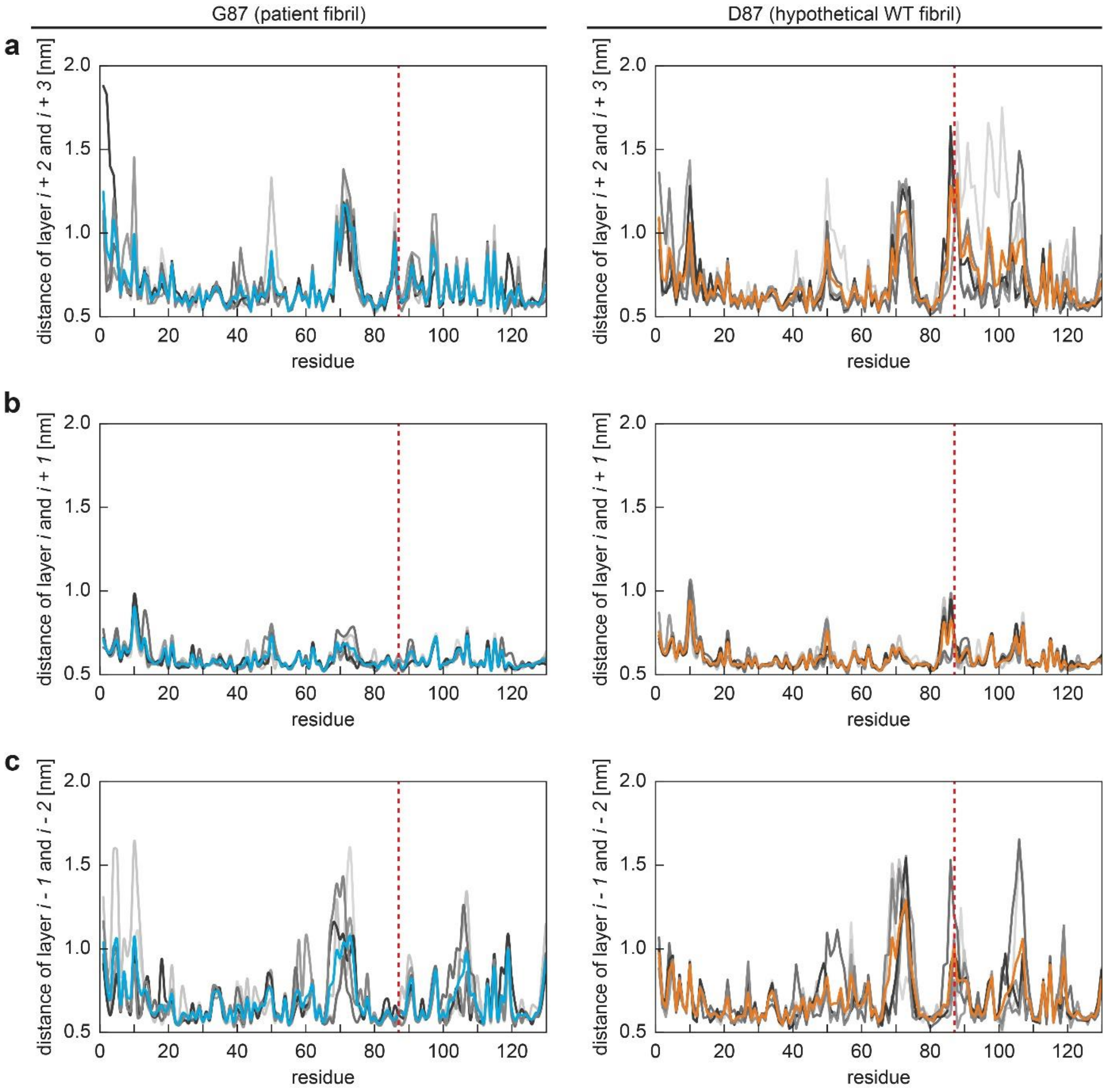

Supplementary Figure 14.

Analysis of the patient fibril and a hypothetical WT fibril with MD simulations.

(a) Maximum distance between corresponding residues from two adjacent layers of the patient fibril (G87, left) and the hypothetical WT fibril with a D87 residue (right), measured between layers $i + 2$ and $i + 3$ from the MD simulation (see Fig. 3b for the definition of the layers). (b) The same as in (a) for layers i and $i + 1$. (c) The same as in (a) for layers $i - 1$ and $i - 2$. Grey: results from individual MD simulations (n=6), blue/orange: mean values. The red dotted lines indicate residue 87.

Supplementary Figure 15

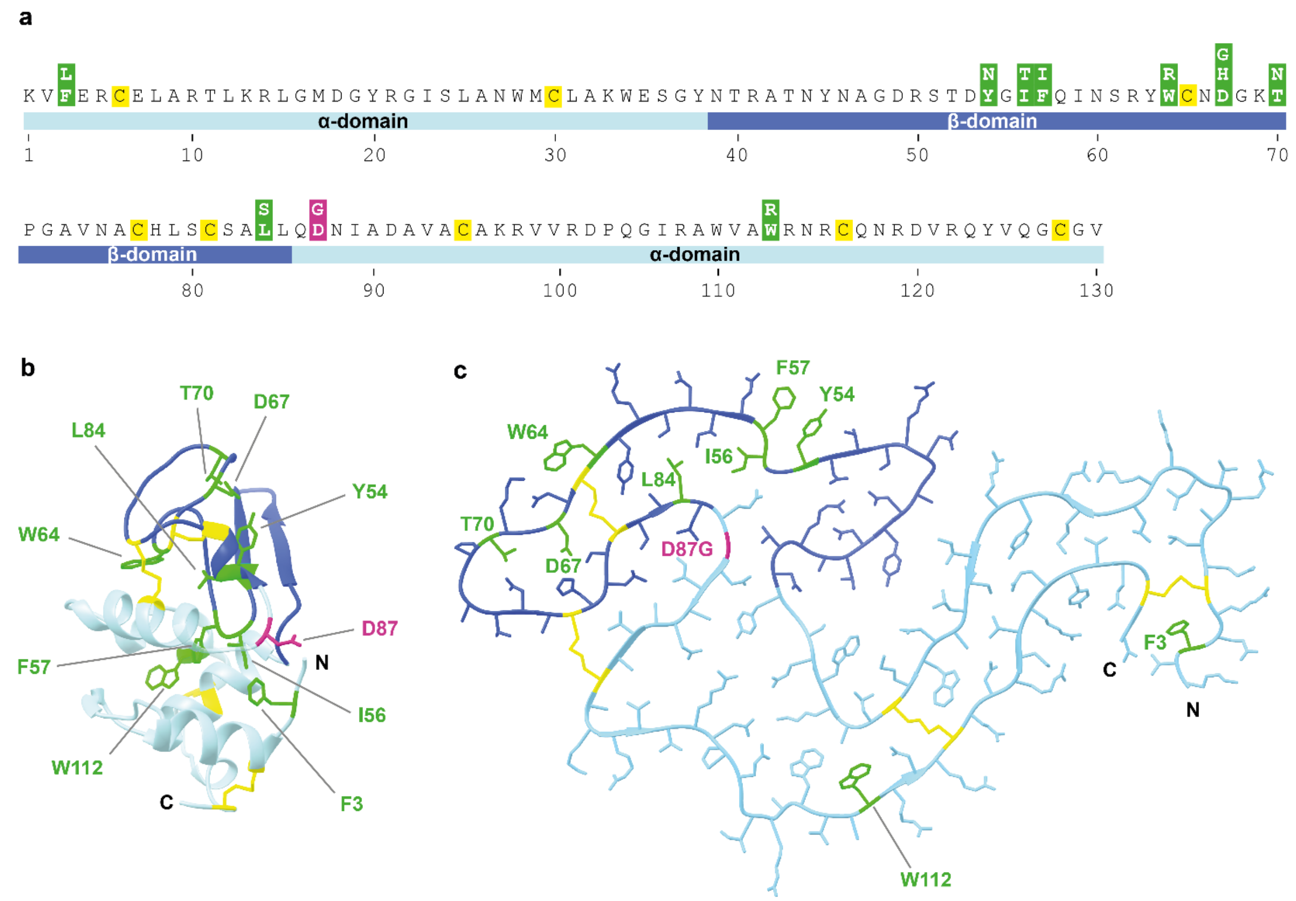

Supplementary Figure 15.

Location of amyloidosis-associated mutations.

(a) Amino acid sequence of human WT lysozyme. Above the sequence: mutational variants associated with ALys amyloidosis. (b) Ribbon diagram of natively folded WT lysozyme (protein data bank: 1JSF [5]). (c) Structural representation of one fibril protein showing the location of the mutational sites. Magenta: mutational site in our fibril; green: previously reported mutations; light blue: α-domain; dark blue: β-domain; yellow: Cys residues forming disulfide bonds. The color code in this figure is kept consistent in all panels.

Supplementary Tables

Supplementary Table 1

**Microscope**	Titan Krios (Thermo Fisher Scientific)
**Camera**	Quantum K2
**Acceleration voltage (kV)**	300
**Magnification**	x 130,000
**Defocus range (µm)**	-1.0 to -2.0
**Dose rate (e^-/Å^2/s)**	6.17
**Number of movie frames**	40
**Exposure time (s)**	8
**Total electron dose (e^-/Å^2)**	49.36
**Pixel size (Å)**	1.04
**Box size (pixel)**	300
**Inter box distance (Å)**	33.5
**Micrographs acquired**	2013
**Number of extracted segments**	120,356
**Number of segments after 2D classification**	120,356
**Number of segments after 3D classification**	120,356
**Resolution, 0.143 FSC criterion (Å)**	2.8
**Map sharpening B-Factor (Å^2)**	-75.9
**Helical rise (Å)**	4.78
**Helical twist (°)**	-1.4
**Imposed symmetry**	C1

Supplementary Table 1.

Data collection and reconstruction parameters.

Supplementary Table 2

**Initial model**	de novo
**Model resolution (Å)**	
FSC threshold 0.143	2.6
FSC threshold 0.5	3.0
**Model composition**	
Non-hydrogen atoms	1,025
Protein residues	130
Ligands	0
**R.m.s. deviations**	
Bond lengths (Å)	0.61
Bond angles (°)	1.03
**Validation**	
MolProbity score	1.71
Clashscore	5.51
Poor rotamers (%)	0.00
**Ramachandran plot**	
Favored (%)	93.75
Allowed (%)	6.25
Disallowed (%)	0
**Map CC**	
CC mask	0.79

Supplementary Table 2.

Modelling parameters.

References

1. Pettersen, E. F. *et al.* UCSF Chimera—A visualization system for exploratory research and analysis. *Journal of Computational Chemistry* **25**, 1605–1612 (2004).
2. Krissinel, E. & Henrick, K. Inference of Macromolecular Assemblies from Crystalline State. *Journal of Molecular Biology* **372**, 774–797 (2007).
3. Sawaya, M. R., Hughes, M. P., Rodriguez, J. A., Riek, R. & Eisenberg, D. S. The expanding amyloid family: Structure, stability, function, and pathogenesis. *Cell* **184**, 4857–4873 (2021).
4. Vanhee, P. *et al.* BriX: a database of protein building blocks for structural analysis, modeling and design. *Nucleic Acids Research* **39**, D435–D442 (2011).
5. Harata, K., Abe, Y. & Muraki, M. Full-matrix least-squares refinement of lysozymes and analysis of anisotropic thermal motion. *Proteins* **30**, 232–243 (1998).
